# Splice-variant specific effects of a *CACNA1H* mutation associated with writer’s cramp

**DOI:** 10.1186/s13041-021-00861-z

**Published:** 2021-09-20

**Authors:** Ivana A. Souza, Maria A. Gandini, Gerald W. Zamponi

**Affiliations:** Department of Physiology and Pharmacology, Hotchkiss Brain Institute, Cumming School of Medicine, Alberta Children’s Hospital Research Institute,, University of Calgary, Alberta Calgary, Canada

**Keywords:** *CACNA1H*, Calcium channel, Ca_v_3.2, Alternative splicing, Splice variant, Writer’s cramp

## Abstract

The *CACNA1H* gene encodes the α1 subunit of the low voltage-activated Ca_v_3.2 T-type calcium channel, an important regulator of neuronal excitability. Alternative mRNA splicing can generate multiple channel variants with distinct biophysical properties and expression patterns. Two major splice variants, containing or lacking exon 26 (± 26) have been found in different human tissues. In this study, we report splice variant specific effects of a Ca_v_3.2 mutation found in patients with autosomal dominant writer’s cramp, a specific type of focal dystonia. We had previously reported that the R481C missense mutation caused a gain of function effect when expressed in Ca_v_3.2 (+ 26) by accelerating its recovery from inactivation. Here, we show that when the mutation is expressed in the short variant of the channel (− 26), we observe a significant increase in current density when compared to wild-type Ca_v_3.2 (− 26) but the effect on the recovery from inactivation is lost. Our data add to growing evidence that the functional expression of calcium channel mutations depends on which splice variant is being examined.

The *CACNA1H* gene encodes the pore-forming α1 subunit of the Ca_v_3.2 calcium channel isoform which, along with Ca_v_3.1 (*CACNA1G*) and Ca_v_3.3 (*CACNA1I*), form the low-voltage activated T-type calcium channel family. The three isoforms generate currents with distinct biophysical and pharmacological properties and help regulate neuronal excitability, hormone secretion and cardiac function. Alternative mRNA splicing and differential splice-variant expression patterns further enhance the functional diversity of T-type channels [1].

Inherited or de novo mutations found in Ca_v_3.2 have been associated with numerous disorders, including epilepsy, primary aldosteronism, pain, autism and amyotrophic lateral sclerosis (for review see [2]). Many of these mutations have been characterized using heterologous expression systems and although a subset of these variants have been shown to cause significant biophysical changes, some produce mild or no alterations of channel function. A few studies have pointed out that mutations may differentially affect the activity of different splice variants, which can partially explain the lack of effects seen in previous reports [3–6]. Another important consideration is that missense, silent and non-coding mutations that do not alter channel function can potentially contribute to disease by disturbing exonic splicing regulatory sites, thus affecting the normal expression of variants [7].

*CACNA1H* has been found to have at least 14 sites for alternative splicing with the potential to generate over 4,000 mRNA transcripts [7]. Two major splice variants, containing or lacking exon 26 (Ca_v_3.2 (± 26)) have been found in multiple human tissues (corresponding to exon 25 in rat that is expressed in roughly half of the channel transcripts in the thalamus [3]). The inclusion of exon 26 adds 6 amino acids to the cytoplasmic domain III-IV linker region of Ca_v_3.2 (Fig. 1a) and can cause changes in the biophysical properties of the channel, including a hyperpolarizing shift in the voltage dependence of activation and slower recovery from inactivation [7, 8]. We have recently shown that a R481C Ca_v_3.2 missense variant that segregated in a family with autosomal dominant-inherited writer’s cramp (WC) alters the biophysical properties of Ca_v_3.2 (± 26) channels. Electrophysiological analysis of R481C cloned into Ca_v_3.2 (+ 26) showed that mutated channels had a significant faster recovery from inactivation when compared to wild-type (WT) Ca_v_3.2, while current density and steady-state inactivation properties remained the same [9]. Here, we tested the effects of this mutation in a channel backbone that lacks the exon 26 sequence in transfected tsA-201 cells. Figure 1b shows representative Ba^2+^ current traces from WT and R481C channels. Different from what was seen in Ca_v_3.2 (+ 26), when R481C was expressed in Ca_v_3.2 (**− **26), we noted a significant increase in current density as shown in the current density-voltage relationship and maximal conductance graphs (Fig. 1c, d). Steady state inactivation and recovery from inactivation properties were not different from WT channels (Fig. 1e–h). It is important to note that while the R481C mutation causes different effects depending on which splice variant is being tested, these effects imply a Ca_v_3.2 gain of function in both exon 26 containing and lacking channels, which can lead to increased neuronal excitability and contribute to a dystonic phenotype [10]. Although unlikely, it is unknown whether patients carrying the R481C mutation may also have alterations in exon 26 splicing that may exacerbate the effect of the mutations in specific tissues.

**Fig. 1:**
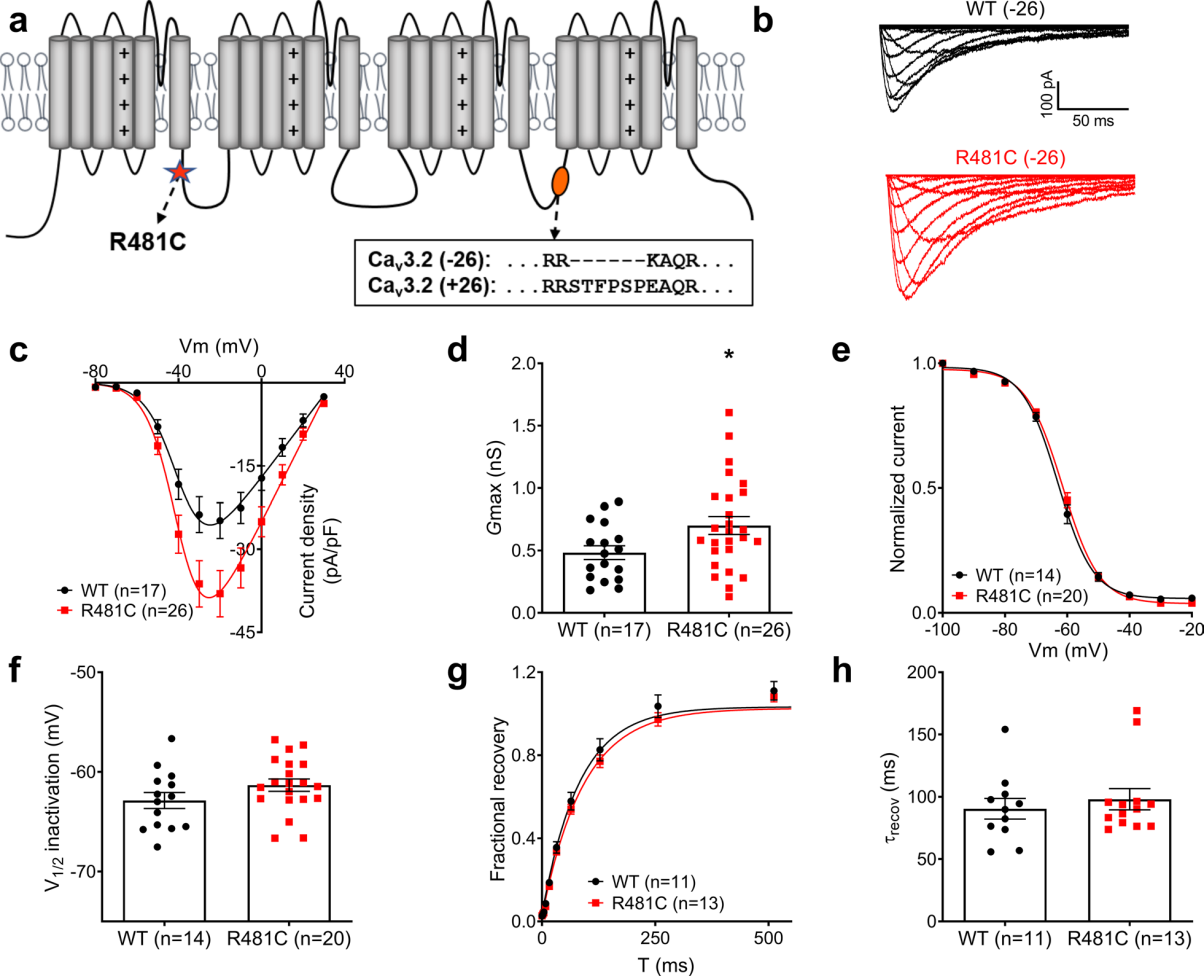
Electrophysiological recordings from tsa-201 cells expressing wild-type and R481C mutant channels lacking exon 26 (Ca_v_3.2 (-26)). **a** Schematic representation of the Ca_v_3.2 channel α1 subunit showing the approximate location of the R481C mutation (I-II linker) and exon 26 (III-IV linker). **b** Representative Ba^2+^ current traces recorded from WT and R481C channels. **c** Average current densities (pA/pF) as a function of voltage showing an approximate 30 % increase in current densities of mutant channels compared to WT. **d** Bar graph representing the corresponding maximum conductance *G*_max_. Values are represented as mean ± SEM. The asterisk denotes statistical significance relative to WT (*p = 0.035, Student’s t-test). **e** Steady-state inactivation curves for WT and R481C channels. **f** Mean half-inactivation potentials obtained from fits with the Boltzmann equation of individual steady-state inactivation curves. **g** Time course of recovery from inactivation for WT and R481C channels. **h** Time constant of recovery from inactivation obtained by individual fits of the recovery from inactivation data

As mentioned above, splice variant specific effects of point mutations have previously been shown for Ca_v_3.2 channels. The R1584P mutation found in the genetic absence epilepsy rats from Strasbourg (GAERS) model only manifests itself functionally when introduced into the Ca_v_3.2 variant that carries exon 26 (exon 25 in rats) [3]. Point mutations associated with primary aldosteronism have also been shown to have splice variant specific effects. Three mutations (S196L, V1951E and P2083L) caused significant changes in Ca_v_3.2 (+ 26) but not in Ca_v_3.2 (-26) channels, while M1549I altered both channel splice variants function [6]. Interestingly, the authors of this study found that human zona glomerulosa cells, which produce the hormone aldosterone, only express the long Ca_v_3.2 (+ 26) channels. Since both splice variants are expressed in the brain, their results can partially explain why only patients with the M1549I mutation have neuronal abnormalities in addition to aldosteronism [6]. Splice variant specific effects of mutations have also been reported in the high-voltage activated Ca_v_2.1 P/Q-type channels. Three type-1 familial hemiplegic migraine (FHM-1) mutations alter channel function differently when expressed in Ca_v_2.1 containing or lacking exon 47 [4]. Our group has also reported an FHM-1 (Y1384C) mutation that has differential effects on recovery from inactivation dependent on which Ca_v_2.1 splice variant (± 47) is being tested [5].

The R481C Ca_v_3.2 mutation has been previously found in a patient with bilateral trigeminal neuralgia [11]. This raises the question as to why identical mutations can generate distinct phenotypes in different patients. In fact, highly penetrant mutations for severe Mendelian diseases have been found in healthy individuals [12]. Considering the number of mRNA transcripts that *CACNA1H* can generate, mutations can potentially produce different spatial and temporal effects depending on splice variant expression patterns. In addition, there is growing evidence suggesting the importance of other genes for the penetrance and expressivity of mutations [13]. Notably, besides the mutation in *CACNA1H*, three additional missense mutations in other genes segregated with disease phenotype in the family affected with writer’s cramp [9]. One of these genes, *SPTBN5*, encodes the protein spectrin-βV, a member of the spectrin family of cytoskeletal proteins. Interestingly, our group has shown that Ca_v_3.2 channels interact and can be modulated by at least three spectrin proteins: spectrin-αII, spectrin-βI and spectrin-βII [14]. Whether the mutation in the *SPTBN5* gene affects the expression of the R481C mutation in *CACNA1H* contributing to the pathophysiology of WC is unknown. Finally, we note that splicing of exon 26 interferes with calnexin-dependent retention of the channel in the ER, thus increasing channel expression at the cell surface [15]. It is possible that in Ca_v_3.2 (-26) channels, there is a synergistic effect between the domain I-II linker mutation and the enhanced ability of calnexin to facilitate ER export.

In conclusion, our study provides further evidence that *CACNA1H* alternative splicing may be important in the pathophysiology of genetic disorders and highlights complexity of the mechanisms by which a mutation can contribute to disease.

## Data Availability

All data generated or analysed during this study are included in this published article.

## Abbreviations

RNA: Ribonucleic acid
mRNA: Messenger ribonucleic acid
WC: Writer’s cramp
WT: Wild-type
GAERS: Genetic absence epilepsy rats from Strasbourg
FHM-1: Type-1 familial hemiplegic migraine
